# Estimating treatment effects in clinical studies of Alzheimer's disease: What are estimands and why do we need them?

**DOI:** 10.1002/alz.71676

**Published:** 2026-08-26

**Authors:** Suzanne Hendrix, Craig Mallinckrodt, Rikke Mortensen Abschneider, Alireza Atri, Mette Dahl Bendtsen, Teresa Leon Colombo, Howard H. Feldman, Peter Johannsen, Jeffrey Cummings

**Affiliations:** ^1^ Pentara Corporation Millcreek Utah USA; ^2^ Novo Nordisk A/S Søborg Denmark; ^3^ Banner Sun Health Research Institute Sun City Arizona USA; ^4^ Banner Alzheimer's Institute Phoenix Arizona USA; ^5^ Brigham and Women's Hospital, Harvard Medical School Boston Massachusetts USA; ^6^ Alzheimer's Disease Cooperative Study, Department of Neurosciences University of California San Diego, La Jolla San Diego California USA; ^7^ Chambers‐Grundy Center for Transformative Neuroscience Department of Brain Health Kirk Kerkorian School of Medicine University of Nevada Las Vegas Nevada USA

**Keywords:** Alzheimer's disease, clinical trials, estimands, semaglutide, treatment effect

## Abstract

In clinical studies, it is scientifically important, and a regulatory expectation, that objectives be translated into key clinical questions by specifically defining treatment effects to be estimated. Estimands are part of a structured framework, as presented in International Council for Harmonisation (ICH) E9(R1), by which study objectives are linked to a suitable study design and tools for estimation. Estimands are constructed using five attributes: treatment, population, variable (or endpoint), population‐level summary for the variable, and intercurrent events (ICEs). ICEs occur after treatment initiation and can affect the existence or interpretation of the measurements. In Alzheimer's disease (AD), potential ICEs include additional AD medication use, discontinuation of treatment, and death. We describe estimands in recent clinical studies of anti‐amyloid therapies, including gantenerumab, lecanemab, and donanemab, in AD and use the evoke and evoke+ studies of semaglutide in early‐stage symptomatic AD as examples of estimand application in AD trials.

## Highlights

- In clinical studies, it is a regulatory expectation and scientifically important that the study objectives be translated into key clinical questions of interest by defining estimands. An estimand is a precise description of the treatment effect to be estimated that reflects the clinical question posed by the study objective.
- Here, we describe estimands, including examples of how they should be used and interpreted in clinical studies of treatments for AD, with particular focus on the estimands used in the evoke and evoke+ studies assessing the use of the glucagon‐like peptide‐1 receptor agonist semaglutide in early‐stage symptomatic AD.

## 1 BACKGROUND

In clinical studies, it is scientifically important and a regulatory expectation that the study objectives be translated into key clinical questions of interest by defining estimands. An estimand is a precise description of the treatment effect to be estimated that reflects the clinical question posed by the study objective. Although relatively new, estimands are an essential part of a structured framework to link study objectives to a suitable design and analyses as outlined in the International Council for Harmonisation (ICH) E9(R1) addendum on estimands and sensitivity analysis in clinical trials.^1^ The study objective and estimand define what to estimate (i.e., the question being asked), while the study design, main estimator, and sensitivity estimator(s) specify how to answer that question. This framework ensures that critical aspects of the statistical analysis plan (SAP) are clearly specified and important questions are considered a priori, avoiding ambiguity in interpreting the results. Despite their central role in trial design and interpretation, estimands remain unfamiliar to many clinicians, which hinders their optimal application and understanding.

Defining the estimand entails a broad drug development approach that involves a multidisciplinary discussion of the clinical questions related to each of the study objectives.^2^, ^3^, ^4^ The choice of estimand affects the study design and conduct as well as the analysis and interpretation of the results.^4^

The estimand framework is mandatory for ongoing and newly initiated clinical studies that will be used for regulatory decision‐making, and study results should be reported accordingly.^5^, ^6^ Additionally, the CONsolidated Standards of Reporting Trials (CONSORT) 2025 statement for reporting randomized trials states that study objectives should be defined in terms of the estimand framework if designed as such.^7^ However, the increasing formalization of estimands through regulatory guidance has not consistently been accompanied by clear and accessible descriptions in the scientific literature.

Regulatory guidance specific to the clinical investigation of treatments for Alzheimer's disease (AD) also states that a specification of the estimand is required.^8^ This guidance is currently being updated, including consideration of the definition of estimands in a setting where amyloid‐targeting treatments may increasingly be used.^9^

Here, we describe estimands, including examples of how they can be used and interpreted in clinical studies of treatments for AD. As an illustration, we present the use of estimands in the evoke and evoke+ studies assessing the use of the glucagon‐like peptide‐1 receptor agonist semaglutide in early‐stage symptomatic AD.

## 2 EVOKE AND EVOKE+ STUDIES

The evoke and evoke+ clinical studies were two randomized, double‐blind, placebo‐controlled phase 3 studies investigating the efficacy, safety, and tolerability of once‐daily oral semaglutide 14 mg versus placebo in early‐stage symptomatic AD.^10^, ^11^ Eligible participants were adults aged 55–85 years with mild cognitive impairment (MCI) or mild dementia due to AD with confirmed amyloid abnormalities. After a maximum 12‐week screening phase, 1855 participants in evoke and 1953 participants in evoke+ were randomized (1:1) to once‐daily oral semaglutide or placebo for a 104‐week main treatment phase and a 52‐week extension phase. The primary endpoint was the change from baseline to week 104 in the Clinical Dementia Rating – Sum of Boxes (CDR‐SB) score. The two studies had identical designs, except that evoke+ allowed the inclusion of patients with evidence of significant small vessel pathology in the brain, defined as more than one lacunar infarct and/or age‐related white‐matter change of > 2 at baseline. Given their identical designs, evoke and evoke+ had identical estimands and analytic approaches, which are discussed below to illustrate the estimand framework.

Results from the evoke and evoke+ studies showed that treatment with oral semaglutide did not slow the rate of progression of early symptomatic AD.^12^

## 3 ESTIMAND FRAMEWORK

### 3.1 Perspectives on choice of estimands

When designing clinical studies, it is important to consider the decisions that will need to be made by various stakeholders based on the evidence that the study is intended to generate. These decisions should be considered when setting the study objectives. For example, regulatory authorities may need to decide if a treatment being tested should be granted marketing authorization. Payers decide if and where a new drug belongs on a formulary list, whether the treatment will be funded, and/or the appropriate reimbursement. Hence, regulators and payers make decisions that influence large and diverse groups of patients and caregivers. These stakeholders are therefore often most interested in “real‐world” treatment effects that include both patients who do and do not adhere to the defined treatment regimen, and who do or do not begin additional medication. In contrast, clinicians and patients make decisions about the treatment of individual patients. These stakeholders may be more interested in understanding the expected treatment effect if the patient adheres to medication for the duration of treatment without additional medication.^2^ Given the differing perspectives of stakeholders, it is often necessary to pre‐specify multiple estimands.

Previously, participants who discontinued treatment may have been withdrawn from the study and their data excluded from analyses; however, treatment discontinuation may have been related to treatment efficacy and/or tolerability, and biased discontinuation may introduce complexities or potential confounds when evaluating the results. Hence, the data collected following treatment discontinuation can be informative for estimating treatment effects. In addition, the handling of data for participants who initiated additional medication is often not clearly specified. Estimands help to clarify and address these issues, with the choice of estimand and the subsequent analyses that are to be performed taking these events into account.

### 3.2 Development of estimands

**FIGURE 1:**
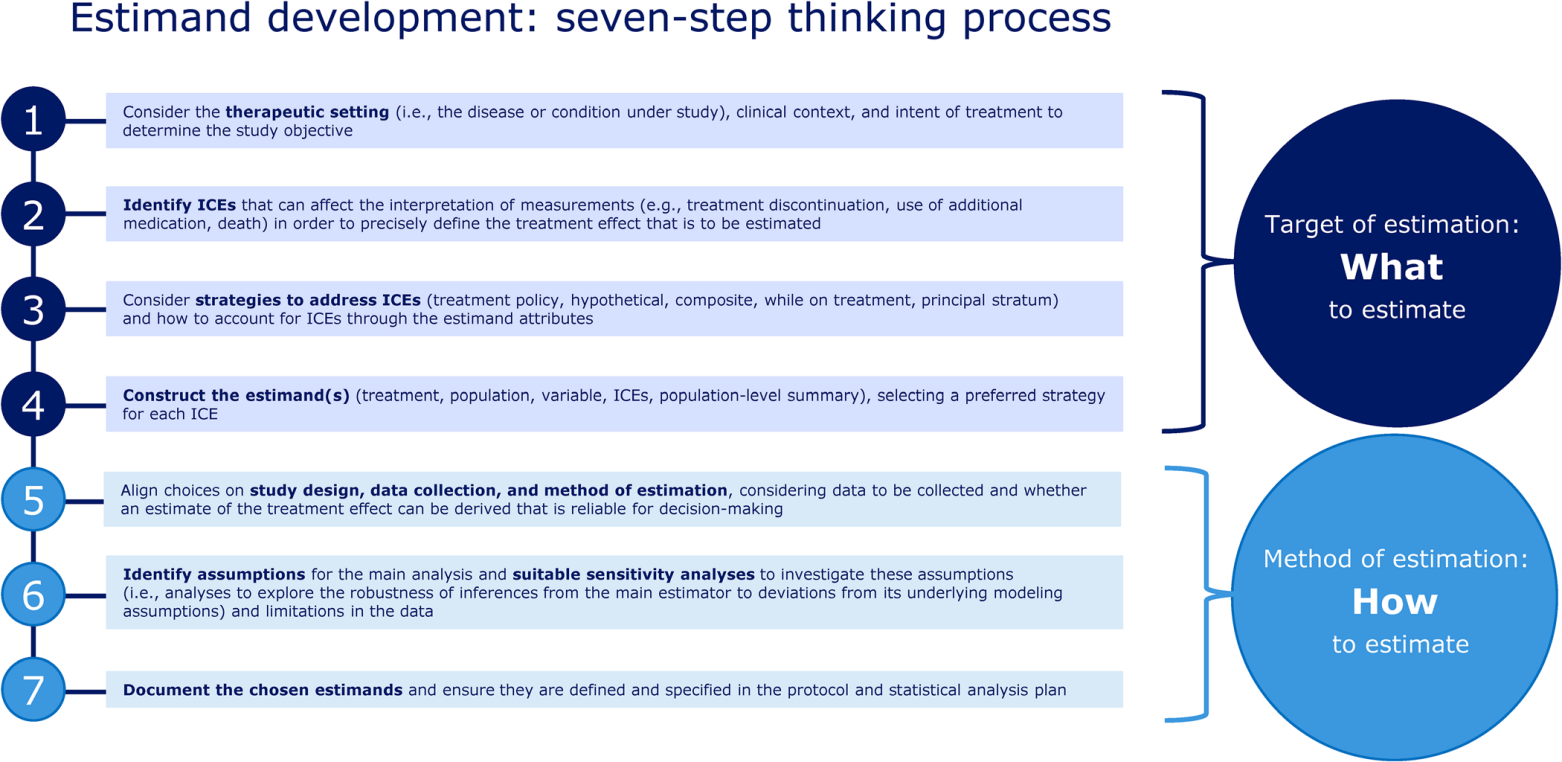
Estimand framework and thinking process. ICE, intercurrent event.

The development of estimands involves a seven‐step thinking process, outlined in ICH E9(R1) training standards^13^ and discussed by Lynggaard et al.^4^ (Figure 1). The first step is to determine the study objective. The second and third steps are to identify intercurrent events (ICEs) (i.e., events that occur after randomization, such as additional medication or treatment discontinuation, that affect the existence or the interpretation of the measurements in the study) and then decide upon strategies to address these ICEs. The fourth step is to construct the primary estimand, with similar steps then followed for secondary estimands if more than one estimand is required. The definition of each estimand should include the five attributes summarized in step 4 below, with a strategy chosen for each ICE. These first four steps clarify the “What” that is to be measured.

Subsequent steps include specification of how to answer the question specified in the first four steps: the “How”. The fifth step involves aligning the study design and the statistical methodology to determine the estimand (treatment effect). In the sixth step, sensitivity analyses aligned to the same estimand as the main analytical approach should be detailed. This includes a specification of which underlying assumption of the main analysis the sensitivity analysis is targeting. The seventh step relates to documenting the estimand.^14^ However, defining the estimands is not necessarily as straightforward as outlined in the seven steps of the thinking process. For evoke and evoke+, there were several interactions with health authorities, and, based on their feedback, the estimands were updated during the studies.

#### 3.2.1 Step 1: Therapeutic setting and intent of treatment to determine a trial objective

The first step is to determine the study objective based on the therapeutic setting (i.e., the disease and clinical context) and intent of treatment. In evoke and evoke+, the objective was to assess the effect of oral semaglutide once daily versus placebo when added to standard of care with respect to changes in cognition and function in patients with MCI or mild dementia due to AD. The consideration of the treatment setting and of the intent of treatment starts to inform the estimand framework.

#### 3.2.2 Steps 2 and 3: Identify and discuss strategies to address ICEs

The second and third steps are to identify ICEs and then formulate strategies to address these ICEs. These strategies are driven by the study objective. The primary considerations in identifying ICEs are whether the event affects either the interpretation or the existence of the measurements associated with the clinical questions of interest.

It is necessary to specify the strategy used to address ICEs in order to precisely define the treatment effect that is to be estimated. In the evoke and evoke+ studies, three ICEs were defined: (1) permanent discontinuation of study treatment, (2) initiation of additional AD medication after randomization (e.g., anti‐amyloid monoclonal antibodies), and (3) death. Permanent discontinuation of study treatment, which may or may not be treatment related, and the use of additional AD medication both affect the interpretation of change in CDR‐SB score, while death will affect the existence of the measurement (i.e., CDR‐SB cannot be assessed after death).

Other examples of potential ICEs in AD studies could include discontinuation of non‐investigational medications (including additional medications permitted in the protocol), use of behavioral treatments, and the occurrence of events not primarily related to AD or the treatment of AD. A participant can experience more than one ICE, and rates of ICEs can be high in AD studies given that populations are often elderly with multiple comorbid medical conditions and frequent use of additional medications.^15^, ^16^

Importantly, ICEs are distinct from missing data, which are defined in ICH E9(R1) as data that would be meaningful for the analysis of a given estimand but were not collected. Missing data (e.g., due to study withdrawal or loss to follow‐up) are not reflected in the estimand but instead represent data limitations that need to be addressed in the statistical analysis.

The European Medicines Agency (EMA) ICH E9(R1) addendum outlines five strategies for handling ICEs in the estimands framework: treatment policy, hypothetical, composite, principal stratum, and while on treatment. These are explained in Table 1. The strategies to be used to handle ICEs should reflect the clinical question of interest being addressed by the study, and each strategy can be implemented in several ways. Different strategies may be used for different ICEs within the same estimand, an approach referred to as a “hybrid strategy”. Hypothetical strategies envisage scenarios in which the ICE does not occur. It is important to note that many potential hypothetical scenarios can be envisaged, but not all will be of interest for regulatory decision‐making.

**TABLE 1:** Strategies for addressing ICEs.

Strategy	Consideration of the ICE
Treatment policy	The occurrence of the ICE is considered irrelevant in defining the treatment effect of interest: the value for the variable of interest is used regardless of whether the ICE occurs.
Hypothetical	A scenario is envisaged in which the ICE would not occur: the value of the variable to reflect the clinical question of interest is the value that the variable would have taken in the hypothetical scenario defined. Many potential hypothetical scenarios can be envisaged, but not all will be of interest for regulatory decision‐making.
Composite	An ICE is considered in itself to be informative about the patient's outcome and is therefore incorporated into the definition of the variable.
While on treatment	Response to treatment prior to the occurrence of the ICE is of interest. If a variable is measured repeatedly, its values up to the time of the ICE may be considered relevant for the clinical question, rather than the value at the same fixed time point for all participants.
Principal stratum	This relates to the population of interest. The target population might be taken to be the principal stratum in which an ICE would occur. Alternatively, the target population might be taken to be the principal stratum in which an ICE would not occur. The clinical question of interest relates to the treatment effect only within the principal stratum.

Abbreviation: ICE, intercurrent event.

#### 3.2.3 Step 4: Construct the estimands

Estimands are constructed using five attributes, with a strategy chosen for each ICE. Components of the estimand framework have largely been familiar to clinical trialists; however, the framework formalizes their interplay, particularly in relation to ICEs, which represent a key concept introduced within this framework.

- **Treatment of interest**: This refers to the intervention being investigated, and the comparator arm; in evoke/evoke+, these were oral semaglutide or placebo. It is important to clearly specify the intended regimen given that many ICEs involve deviations from the intended treatment. Frequent considerations for AD trials include whether additional AD medication is part of the regimen of interest and whether data after discontinuation of study drug is of interest.
- **Population**: This is the population for which inferences are sought, or in the context of regulatory decision‐making, the population for which the treatment is indicated in its label. Examples in AD include patients with early AD or mild‐to‐moderate AD. In evoke/evoke+, this was patients with MCI or mild dementia due to AD, with amyloid positivity required by trial inclusion criterion.
- **Variable or endpoint**: This is the variable (or endpoint) required to address the clinical question. In evoke/evoke+, the primary endpoint was change in CDR‐SB score from baseline to week 104.
- **Population‐level summary measure**: This is a statistical measure that provides the basis for comparison between treatments. If applicable, the specific time point of interest should be part of the definition. A common example in AD trials is the difference between treatments in mean change in trial endpoint, for example, CDR‐SB score, from baseline to end of treatment duration.
- **ICE**: ICEs and strategies for handling them have been described in Steps 2 and 3 (see Section 3.2.2). Precise definitions of treatment, population, and variable are essential when constructing an estimand, and these attributes often determine how some ICEs are handled. For example, in the evoke/evoke+ treatment policy estimand, permanent treatment discontinuation and use of additional AD medication were addressed through the treatment condition. If no strategy is specified for other ICEs (ICEs not captured by the other attributes), they are usually handled implicitly by a treatment policy approach, that is, by analyzing the available data as collected, after the event has occurred.

**FIGURE 2:**
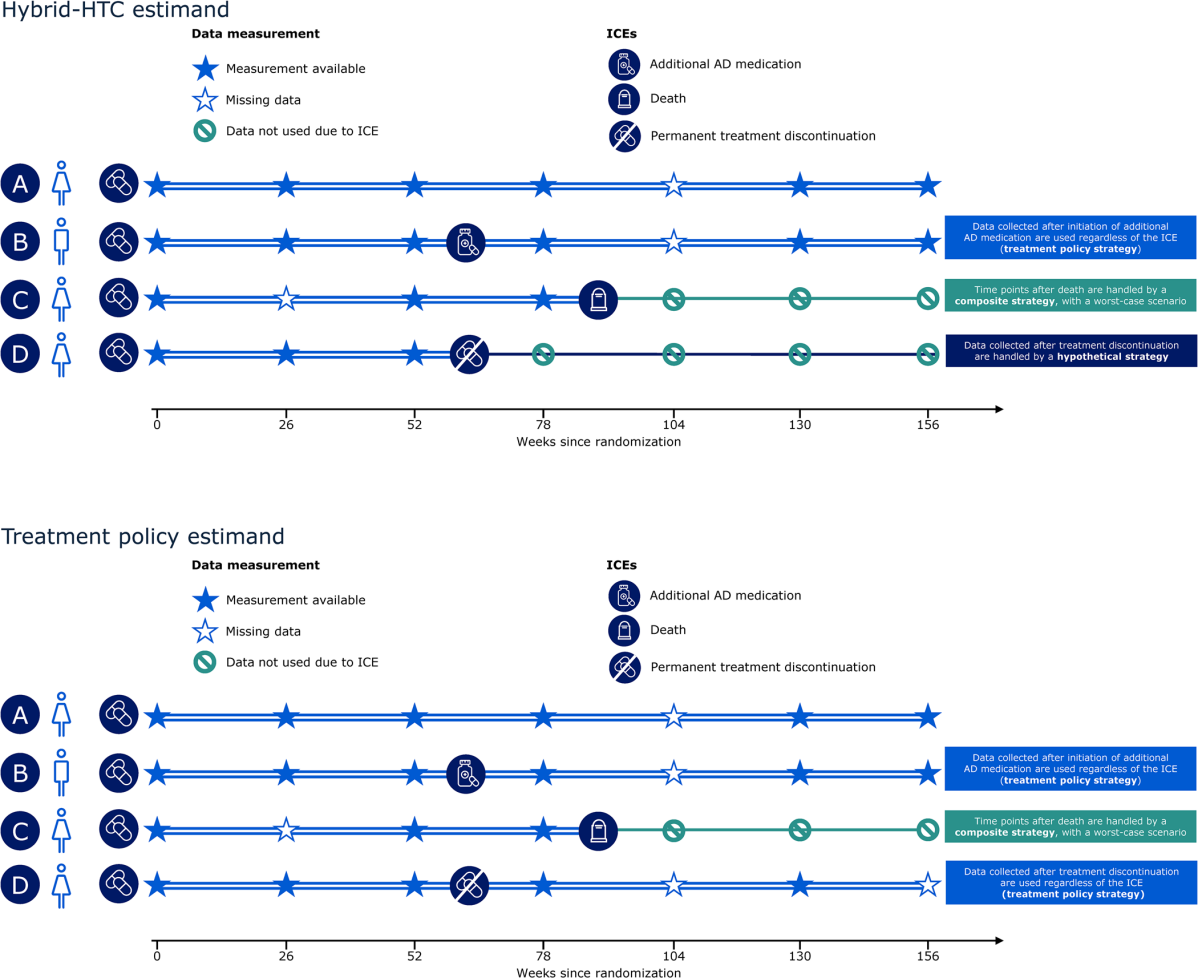
Hybrid‐HTC and treatment policy estimands. AD, Alzheimer's disease; HTC, hypothetical, treatment policy, and composite; ICE, intercurrent event.

In evoke and evoke+, two primary estimands were defined (Table 2). These two estimands address two different clinical questions to serve the diverse needs of stakeholders that together provide a broad understanding of the treatment effect. The first estimand was designated the hybrid‐hypothetical, treatment policy, and composite (HTC) estimand, which aimed to answer the question: “What is the difference in the mean change in the CDR‐SB score from baseline to week 104 in the participating population of patients with MCI or mild dementia due to AD, comparing oral semaglutide once daily to placebo, assuming that patients remained on treatment, regardless of additional AD medication use, and considering death as a worst‐case outcome?” (Figure 2). This was designated the hybrid‐HTC estimand because different ICEs in the estimand were handled using different strategies, ignoring initiation of additional AD medication (treatment policy strategy), while regarding death as a treatment failure (worst‐case scenario). The hybrid‐HTC estimand was regarded as the primary estimand for all purposes other than for regulatory agencies, for which it was regarded as an additional estimand. The second estimand was designated the treatment policy estimand, which aimed to answer the question: “What is the difference in the mean change of the CDR‐SB score from baseline to week 104 in the participating population of patients with MCI or mild dementia due to AD, comparing oral semaglutide once daily to placebo, regardless of treatment discontinuation or initiation of additional AD medication, and considering death as a treatment failure (worst‐case outcome)” (Figure 2). This is named the “treatment policy estimand” because most of the ICEs are accounted for by a treatment policy strategy, even though death was addressed using a composite strategy. Data collected after the ICEs of treatment discontinuation or initiation of additional AD medication were aligned with a treatment policy strategy (i.e., disregarding the occurrence of the ICE) and data on CDR‐SB collected after the occurrence of these events were used when estimating the treatment effect. Death, regardless of cause and whether related to AD, was handled by a composite strategy, with a worst‐case scenario assigned as the outcome. The treatment policy estimand was regarded as the primary estimand for regulatory agencies and as an additional estimand for all other purposes.

**TABLE 2:** Primary estimands used in evoke and evoke+, defined from the five elements outlined in ICH E9(R1).

Clinical question	Estimand type	Attributes				
		Treatment condition	Endpoint	Population	ICEs^a^	Population‐level summary measure
What is the difference in mean change in CDR‐SB score from baseline to week 104 in patients with MCI or mild dementia due to AD, with oral semaglutide OD vs. placebo, if the patients had remained on IMP, regardless of use of additional AD medication, and assuming death as a worst‐case outcome?	Hybrid‐HTC	IMP; had all participants remained on IMP, regardless of use of additional AD medication	Change in CDR‐SB score from baseline to week 104	Patients with MCI or mild dementia due to AD	Hypothetical strategy for permanent treatment discontinuationsTreatment policy strategy for use of additional AD medicationComposite strategy for death	The difference in mean change from baseline
What is the difference in mean change in CDR‐SB score from baseline to week 104 in patients with MCI or mild dementia due to AD, with oral semaglutide OD vs. placebo, regardless of treatment discontinuation and/or use of additional AD medication, and assuming death as a worst‐case outcome?	Treatment policy	IMP regardless of permanent treatment discontinuation and/or use of additional AD medication			Treatment policy strategy for permanent treatment discontinuations, and use of additional AD medicationComposite strategy for death	

Abbreviations: AD, Alzheimer's disease; CDR‐SB, Clinical Dementia Rating–Sum of Boxes; HTC, hypothetical, treatment, and composite strategy; ICE, intercurrent event; ICH, International Council for Harmonisation; IMP, investigational medicinal product; MCI, mild cognitive impairment; OD, once daily.

^a^ All other ICEs are implicitly handled by a treatment policy strategy.

#### 3.2.4 Step 5: Align choices on trial design, data collection, and method of estimation

The fifth step in constructing estimands includes the specification of how to answer the clinical question specified in the first four steps. This involves aligning the study design and the statistical methodology to determine the estimand (treatment effect) and involves consideration of the data to be collected and whether an estimate of the treatment effect can be derived that is reliable for decision‐making. For example, in evoke and evoke+, efforts were made to keep participants in the study (attending visits and undergoing planned assessments) even if they had discontinued randomized treatment. This was specified as part of the trial design to allow for appropriate estimation of the treatment policy estimand, in which treatment discontinuations were handled by a treatment policy strategy.

The approach taken in the evoke and evoke+ studies aligned with the strategies for handling ICEs defined in the estimands. Briefly, for the treatment policy estimand, all available CDR‐SB measurements were included, and missing week 104 values were imputed using a pattern‐mixture multiple imputation approach that accounted for potentially different patterns of disease progression depending on whether participants were on active drug or placebo and whether they had permanently discontinued their study drug. For example, for a participant in the active arm who prematurely discontinued treatment, missing week 104 data were imputed from participants in the active arm who also prematurely discontinued treatment but stayed in study as intended. Further details are provided in the trial protocol and SAP, which have previously been published.^12^

For the hybrid‑HTC estimand, all measurements collected after permanent treatment discontinuation were excluded, and a mixed model for repeated measures was applied, consistent with the hypothetical strategy for treatment discontinuation.

For both estimands, deaths were handled via a composite strategy by imputing missing data following death by a worst‑case outcome equal to the 90th percentile worst observed CDR‑SB value, that is, the imputed value for each participant was calculated by adding the 90th percentile of observed change in CDR‐SB scale (across treatment arms) from baseline up to and including the visit in scope to the CDR‐SB baseline value for the participant, or the max CDR‐SB value of 18 if the calculated number exceeds 18.

#### 3.2.5 Step 6: Identify assumptions for the main analysis and suitable sensitivity analyses to investigate these assumptions

In the sixth step of constructing estimands, sensitivity analyses aligned to the same estimand as the main analytical approach are specified. This includes a specification of which underlying assumption of the main analysis the sensitivity analysis is targeting. For the evoke and evoke+ studies, sensitivity analyses included two‑way tipping‐point analyses to test the robustness of primary analyses to missing‐data assumptions (e.g., for the treatment policy estimand, specifically targeting the missing‐at‐random assumption within each of the four imputation groups). A tipping‐point analysis systematically varies the missing‐data assumptions to identify combinations of deviations from the assumptions behind the primary analysis that would change the conclusion of the analysis (e.g., for the treatment policy estimand, by adding penalties to multiply imputed values in the primary analysis to find the point at which the primary conclusion would change).^17^

#### 3.2.6 Step 7: Document the chosen estimands

The seventh step relates to documenting the chosen estimands in the study protocol and statistical analysis plan. In addition, the primary estimand should be defined in manuscripts that detail the results of a clinical trial.

## 4 ESTIMANDS IN OTHER RECENT AD STUDIES

A variety of approaches have been pursued in defining estimands in neurologic disorders and, more specifically, in clinical studies of AD. A systematic search of the EMA scientific advice database for strategies to address ICEs in confirmatory central nervous system studies found that a treatment policy strategy was the type frequently recommended to address treatment discontinuation and use of additional medication (i.e., data used regardless of whether or not the ICE occurred).^18^ However, other strategies (hypothetical, composite, while on treatment, or principal stratum) were also proposed in some studies. A variety of strategies have been endorsed for the ICE of death, depending on the study setting. Applied to AD, if death was related to the underlying disease, the composite strategy was the preferred choice of regulators.

In AD, there have been modeling and simulation studies, with few examples of estimands being used in registrational clinical trials. Simulation studies that align treatment policy estimands and estimators have addressed the impact of ICEs, sensitivity analyses, and treatment discontinuations.^19^ The impact of the use of symptomatic medications and potential importance of including initiation of symptomatic treatment within an estimand framework has been modeled across scenarios, demonstrating the potential bias of its exclusion.^20^

In two phase 3 studies of gantenerumab (GRADUATE I and II) in participants with MCI or mild dementia due to AD and evidence of amyloid plaques, ICEs were defined as study drug or condition related (SDCR) (i.e., discontinuation of study treatment due to lack of efficacy; due to a safety or tolerability reason; due to use or initiation of protocol‐prohibited medication; or due to any other SDCR‐related ICE) and those that are not study drug or condition related (NSDCR) (i.e., withdrawal from study treatment or substantial reduction in drug exposure due to the COVID‐19 pandemic; discontinuation of study treatment due to purely administrative reasons; or death).^21^ For the primary estimand in GRADUATE I and II, a treatment policy approach was used for all SDCR ICEs, whereas all NSDCR ICEs were handled using a hypothetical approach, with missing data imputed with the standard missing‐at‐random assumption.

Two other recent phase 3 studies also reported using a treatment policy strategy. The phase 3 CLARITY AD study of lecanemab in early AD reported that all observed data were included in the primary analysis, including data collected after ICEs (i.e., initiation of new or change of AD concomitant treatment or treatment discontinuation).^22^ No detailed description of estimand attributes is provided, nor of the strategy for handling ICEs. The methods suggest that three ICEs were identified (initiation of new AD concomitant treatment, change of AD concomitant treatment, and treatment discontinuation) and that all observed data were included regardless of these events, as a proposed estimator for a treatment policy strategy. The phase 3 TRAILBLAZER‐ALZ 2 study of donanemab in early symptomatic AD defined two ICEs (initiation or change to standard‐of‐care medications and discontinuation of donanemab), with both addressed by the intention‐to‐treat principle, which we interpret as a treatment policy strategy.^23^ Other phase 3 studies in AD published since 2020 (of masitinib, crenezumab, sodium oligomannate, atabecestat, and lanabecestat) did not include any reference to estimands.^24^, ^25^, ^26^, ^27^, ^28^

The use of estimands in AD studies is a relatively recent development and is still evolving. The multidimensional nature of choosing a strategy for an ICE for a study of AD disease‐modifying treatment means that choices are dependent on the study objective, underlying disease, therapeutic context, and frequency and timing of ICEs and their relatedness to the disease or treatment.

## 5 CONCLUSION

Regulatory requirements for the conduct, analysis, and reporting of clinical studies have evolved in recent years. Estimands provide a structured description of the treatment effect a study intends to quantify, and the estimand framework ensures consistency and alignment of study objectives, design, and conduct, including data collection, statistical analysis, and interpretation of results. The multidisciplinary seven‐step thinking process is a valuable method for helping define estimands, and the use of the estimand framework provides cross‐functional clarity when interpreting and communicating the estimated treatment effect. It is important to know the estimands being used when comparing treatment effect estimates across studies, to ensure a like‐for‐like comparison. Therefore, an understanding of estimands and how to interpret treatment effects is critical to thoroughly evaluate and compare the results of clinical studies. Learnings from the evoke and evoke+ trials demonstrate that, within AD research, the use of estimands is still evolving. More clarity on reporting estimands in clinical study publications, including in AD, is needed. A consensus for estimands to be used in AD studies of disease‐modifying treatments for different development stages will be welcomed.

## CONFLICT OF INTEREST STATEMENT

Suzanne Hendrix is CEO of Pentara Corporation, and declares no other conflicts of interest. Craig Mallinckrodt is Distinguished Biostatistician at Pentara Corporation, and declares no other conflicts of interest. Alireza Atri has, in the past 10 years, served as a consultant or received honoraria or support for consulting; participating in independent data safety monitoring boards; providing educational lectures, programs and materials; and serving on advisory boards for AbbVie, Acadia, Allergan, Alzheimer's Disease International, the Alzheimer's Association, AriBio, Axovant, Axsome, AZTherapies, Biogen, Eisai, Grifols, Harvard Medical School Graduate Continuing Education, JOMDD, Johnson & Johnson, Life Molecular Imaging/Lantheus, Lundbeck, Merck, Michael J. Fox Foundation, Novo Nordisk, ONO, Otsuka, Prothena, Qynapse, Roche/Genentech, Sunovion, Suven, Synexus, and Vaxxinity. He receives book royalties from Oxford University Press for a medical book on dementia. He receives institutional research grant/contract funding from National Institute on Aging/National Institutes of Health (NIA/NIH; 1P30AG072980), NIA/NIH U24AG057437, 1P30AG072980, R01AG070883, R01AG086363, U01AG082350, U24AG057437), AZ DHS (CTR040636), Foundation for the National Institutes of Health NIH (FNIH), Washington University in St. Louis, Michael J. Fox Foundation, and Gates Ventures. His institution receives/received funding for clinical trial grants, contracts, and projects from government, consortia, foundations, and companies, for which he serves/served as a contracted site principal investigator. He has received/receives honoraria from Novo Nordisk for consulting activities, including for service on the evoke(+) program Steering Committee. Howard H. Feldman discloses a consulting service agreement with Novo Nordisk as well as travel support with all funds paid to UC San Diego. He serves as member of the Steering Committee for the evoke and evoke+ trials. Other disclosures include his receiving grants for UC San Diego from Allyx Therapeutics and Vivoryon Therapeutics (Probiodrug). He also holds service agreements through UC San Diego for consulting with Biosplice Therapeutics, Arrowhead Pharmaceuticals, Axon Neuroscience, and LuMind Foundation. He provides service as a member of data and safety monitoring boards for Janssen Research & Development and Roche/Genentech and is a Scientific Advisory Board member for the Tau Consortium through a UC San Diego service agreement. He has received travel support from Royal Society of Canada, Translating Research in Elder Care (TREC), Association for Frontotemporal Degeneration (AFTD), Rainwater Charitable Foundation, Banner Health, Invictus, and Summeet, and philanthropic support for Alzheimer's therapeutic research through the Epstein Family Alzheimer's Research Collaboration. He receives personal funds for Detecting and Treating Dementia (Serial Number 12/3‐ 2691 U.S. Patent No. PCT/US2007/07008, Washington DC, U.S. Patent and Trademark Office). Jeffrey Cummings has provided consultation to Acadia, Alkermes, ALZpath, Annovis Bio, Artery, Axsome, Biogen, Bristol Myers Squibb, Eisai, Fosun, GAP Foundation, Hummingbird Diagnostics, IGC, Janssen, Julius Clinical, Kinoxis, Lilly, LSP/EQT, Merck (Merck, Sharp, and Dohme), MoCA Cognition, Novo Nordisk, NSC Therapeutics, Otsuka, ReMYND, Roche, Scottish Brain Sciences, Signant Health, Simcere, Sinaptica, and T‐Neuro pharmaceutical, assessment, and investment companies. He is supported by NIGMS grant P20GM109025; NIA R35AG71476; NIA R25AG083721; NINDS RO1NS139383; Alzheimer's Drug Discovery Foundation (ADDF); Ted and Maria Quirk Endowment; Joy Chambers‐Grundy Endowment. Rikke Mortensen Abschneider, Mette Dahl Bendtsen, Teresa Leon Colombo, and Peter Johannsen are full‐time employees and minor shareholders of Novo Nordisk A/S. Author disclosures are available in the Supporting Information.

## Supporting information



Supporting Information

## References

[1] Accessed September 2025. International Council for Harmonisation of Technical Requirements for Pharmaceuticals for Human Use . ICH harmonised guideline: addendum on estimands and sensitivity analysis in clinical trials to the guideline on statistical principles for clinical trials, E9(R1). 2019. https://database.ich.org/sites/default/files/E9‐R1_Step4_Guideline_2019_1203.pdf

[2] Mallinckrodt C , Molenberghs G , Rathmann S . Choosing estimands in clinical trials with missing data. Pharm Stat. 2017;16(1):29‐36. doi:10.1002/pst.1765 27492760

[3] Ratitch B , Bell J , Mallinckrodt C , et al. Choosing estimands in clinical trials: putting the ICH E9(R1) into practice. Ther Innov Regul Sci. 2020;54(2):324‐341. doi:10.1007/s43441-019-00061-x 32072573

[4] Lynggaard H , McKendrick S , Baird M , et al. Applying the estimand framework to clinical pharmacology trials with a case study in bioequivalence. Br J Clin Pharmacol. 2025;91(2):310‐324. doi:10.1111/bcp.16347 39587445 PMC11773105

[5] Accessed September 2025. European Medicines Agency . ICH E9(R1) addendum on estimands and sensitivity analysis in clinical trials to the guideline on statistical principles for clinical trials. Step 5. 2020. https://www.ema.europa.eu/en/documents/scientific‐guideline/ich‐e9‐r1‐addendum‐estimands‐and‐sensitivity‐analysis‐clinical‐trials‐guideline‐statistical‐principles‐clinical‐trials‐step‐5_en.pdf

[6] Accessed May 2025. Food and Drug Administration . E9(R1) statistical principles for clinical trials: addendum: estimands and sensitivity analysis in clinical trials. 2021. https://www.fda.gov/media/148473/download

[7] Hopewell S , Chan AW , Collins GS , et al. CONSORT 2025 statement: updated guideline for reporting randomized trials. JAMA. 2025;333(22):1998‐2005. doi:10.1001/jama.2025.4347 40228499

[8] Accessed May 2025. European Medicines Agency . Guideline on the clinical investigation of medicines for the treatment of Alzheimer's disease. 2018. https://www.ema.europa.eu/en/documents/scientific‐guideline/guideline‐clinical‐investigation‐medicines‐treatment‐alzheimers‐disease‐revision‐2_en.pdf

[9] Accessed September 2025. European Medicines Agency . Concept paper on the need for revision of the guideline on the clinical investigation of medicines for the treatment of Alzheimer's disease. 2025. https://www.ema.europa.eu/en/documents/scientific‐guideline/concept‐paper‐need‐revision‐guideline‐clinical‐investigation‐medicines‐treatment‐alzheimers‐disease_en.pdf

[10] Cummings JL , Atri A , Feldman HH , et al. evoke and evoke+: design of two large‐scale, double‐blind, placebo‐controlled, phase 3 studies evaluating efficacy, safety, and tolerability of semaglutide in early‐stage symptomatic Alzheimer's disease. Alzheimers Res Ther. 2025;17(1):14. doi:10.1186/s13195-024-01666-7 39780249 PMC11708093

[11] Scheltens P , Atri A , Feldman HH , et al. Baseline characteristics from evoke and evoke+: two phase 3 randomized placebo‐controlled trials of semaglutide in participants with early‐stage symptomatic Alzheimer's disease. Alzheimers Dement (N Y). 2026;12(1):e70200. doi:10.1002/trc2.70200 41522368 PMC12789876

[12] Cummings JL , Atri A , Sano M , et al. Efficacy and safety of oral semaglutide 14 mg (flexible dose) in early‐stage symptomatic Alzheimer's disease (evoke and evoke+): two phase 3, randomised, placebo‐controlled trials. Lancet. 2026;407(10544):2167‐2179. doi:10.1016/S0140-6736(26)00459-9 41865758

[13] Accessed October 2025. International Council for Harmonisation of Technical Requirements for Pharmaceuticals for Human Use. ICH E9(R1) estimands and sensitivity analysis in clinical trials. Training material. 2021. https://database.ich.org/sites/default/files/E9%28R1%29%20Training%20Material%20‐%20PDF_0.pdf

[14] Lanius V , Glocker B , Lösch C , et al. Realizing the benefits of the estimand framework when reporting and communicating clinical trial results‐some recommendations. Trials. 2025;26(1):241. doi:10.1186/s13063-025-08915-6 40640873 PMC12247363

[15] Reitz C , Mayeux R . Alzheimer disease: epidemiology, diagnostic criteria, risk factors and biomarkers. Biochem Pharmacol. 2014;88(4):640‐651. doi:10.1016/j.bcp.2013.12.024 24398425 PMC3992261

[16] Lanctôt KL , Hviid Hahn‐Pedersen J , Eichinger CS , et al. Burden of illness in people with Alzheimer's disease: a systematic review of epidemiology, comorbidities and mortality. J Prev Alzheimers Dis. 2024;11(1):97‐107. doi:10.14283/jpad.2023.61 38230722 PMC10225771

[17] O'Kelly M , Ratitch B . Clinical Trials with Missing Data: A Guide for Practitioners. John Wiley & Sons; 2014.

[18] Mészáros L , Lasch F , Delafont B , Guizzaro L . Estimands in CNS trials—a review of strategies for addressing intercurrent events. Contemp Clin Trials Commun. 2024;38:101266. doi:10.1016/j.conctc.2024.101266 38380344 PMC10878841

[19] Polverejan E , Dragalin V . Aligning treatment policy estimands and estimators—a simulation study in Alzheimer's disease. Stats Biopharm Res. 2020;12:142‐154. doi:10.1080/19466315.2019.1689845

[20] Lasch F , Guizzaro L , Pétavy F , Gallo C . A simulation study on the estimation of the effect in the hypothetical scenario of no use of symptomatic treatment in trials for disease‐modifying agents for Alzheimer's disease. Stats Biopharm Res. 2023;15:386‐399. doi:10.1080/19466315.2022.2055633

[21] Bateman RJ , Smith J , Donohue MC , et al. Two phase 3 trials of gantenerumab in early Alzheimer's disease. N Engl J Med. 2023;389(20):1862‐1876. doi:10.1056/NEJMoa2304430 37966285 PMC10794000

[22] van Dyck CH , Swanson CJ , Aisen P , et al. Lecanemab in early Alzheimer's disease. N Engl J Med. 2023;388(1):9‐21. doi:10.1056/NEJMoa2212948 36449413

[23] Sims JR , Zimmer JA , Evans CD , et al. Donanemab in early symptomatic Alzheimer disease: the TRAILBLAZER‐ALZ 2 randomized clinical trial. JAMA. 2023;330(6):512‐527. doi:10.1001/jama.2023.13239 37459141 PMC10352931

[24] Dubois B , López‐Arrieta J , Lipschitz S , et al. Masitinib for mild‐to‐moderate Alzheimer's disease: results from a randomized, placebo‐controlled, phase 3, clinical trial. Alzheimers Res Ther. 2023;15(1):39. doi:10.1186/s13195-023-01169-x 36849969 PMC9972756

[25] Ostrowitzki S , Bittner T , Sink KM , et al. Evaluating the safety and efficacy of crenezumab vs placebo in adults with early Alzheimer disease: two phase 3 randomized placebo‐controlled trials. JAMA Neurol. 2022;79(11):1113‐1121. doi:10.1001/jamaneurol.2022.2909 36121669 PMC9486635

[26] Xiao S , Chan P , Wang T , et al. A 36‐week multicenter, randomized, double‐blind, placebo‐controlled, parallel‐group, phase 3 clinical trial of sodium oligomannate for mild‐to‐moderate Alzheimer's dementia. Alzheimers Res Ther. 2021;13(1):62. doi:10.1186/s13195-021-00795-7 33731209 PMC7967962

[27] Sperling R , Henley D , Aisen PS , et al. Findings of efficacy, safety, and biomarker outcomes of atabecestat in preclinical Alzheimer disease: a truncated randomized phase 2b/3 clinical trial. JAMA Neurol. 2021;78(3):293‐301. doi:10.1001/jamaneurol.2020.4857 33464300 PMC7816119

[28] Wessels AM , Tariot PN , Zimmer JA , et al. Efficacy and safety of lanabecestat for treatment of early and mild Alzheimer disease: the AMARANTH and DAYBREAK‐ALZ randomized clinical trials. JAMA Neurol. 2020;77(2):199‐209. doi:10.1001/jamaneurol.2019.3988 31764959 PMC6902191

