# Supplementary material for: Estimating treatment effects in clinical studies of Alzheimer's disease: What are estimands and why do we need them?
Source: Alzheimers Dement. 2026 Aug 26;22(8):e71676. doi: 10.1002/alz.71676 (PMC13508584; doi:10.1002/alz.71676)
Supplement: Supplementary file 1 [file ALZ-22-e71676-s001.pdf]

ICMJE DISCLOSURE FORM

Date: 01 June 2026
Your Name: Suzanne Hendrix
Manuscript Title: Estimating treatment effects in clinical studies of Alzheimer's disease: what are estimands and why do we need them?
Manuscript number (if known): ADJ-D-26-00566

In the interest of transparency, we ask you to disclose all relationships/activities/interests listed below that are related to the content of your manuscript. "Related" means any relation with for-profit or not-for-profit third parties whose interests may be affected by the content of the manuscript. Disclosure represents a commitment to transparency and does not necessarily indicate a bias. If you are in doubt about whether to list a relationship/activity/interest, it is preferable that you do so.

The following questions apply to the author's relationships/activities/interests as they relate to the current manuscript only.

The author's relationships/activities/interests should be defined broadly. For example, if your manuscript pertains to the epidemiology of hypertension, you should declare all relationships with manufacturers of antihypertensive medication, even if that medication is not mentioned in the manuscript.

In item #1 below, report all support for the work reported in this manuscript without time limit. For all other items, the time frame for disclosure is the past 36 months.

		**Name all entities with whom you have this relationship or indicate none (add rows as needed)**	**Specifications/Comments (e.g., if payments were made to you or to your institution)**
**Time frame: Since the initial planning of the work**			
1	All support for the present manuscript (e.g., funding, provision of study materials, medical writing, article processing charges, etc.) **No time limit for this item.**	Novo Nordisk, A/S	Novo Nordisk funding of medical writing
**Time frame: past 36 months**			
2	Grants or contracts from any entity (if not indicated in item #1 above).	__X _None	
3	Royalties or licenses	__X _None	
4	Consulting fees	__X _None	

5	Payment or honoraria for lectures, presentations, speakers bureaus, manuscript writing or educational events	__X _None	
6	Payment for expert testimony	__X _None	
7	Support for attending meetings and/or travel	__X _None	
8	Patents planned, issued or pending	__X _None	
9	Participation on a Data Safety Monitoring Board or Advisory Board	__X _None	
10	Leadership or fiduciary role in other board, society, committee or advocacy group, paid or unpaid	__X _None	
11	Stock or stock options	__X _None	
12	Receipt of equipment, materials, drugs, medical writing, gifts or other services	__X _None	
13	Other financial or non-financial interests	Pentara Corporation	CEO

Please place an "X" next to the following statement to indicate your agreement:

X I certify that I have answered every question and have not altered the wording of any of the questions on this form.

ICMJE DISCLOSURE FORM

Date: 01 June 2026
Your Name: Craig Mallinckrodt
Manuscript Title: Estimating treatment effects in clinical studies of Alzheimer's disease: what are estimands and why do we need them?
Manuscript number (if known): ADJ-D-26-00566

In the interest of transparency, we ask you to disclose all relationships/activities/interests listed below that are related to the content of your manuscript. "Related" means any relation with for-profit or not-for-profit third parties whose interests may be affected by the content of the manuscript. Disclosure represents a commitment to transparency and does not necessarily indicate a bias. If you are in doubt about whether to list a relationship/activity/interest, it is preferable that you do so.

The following questions apply to the author's relationships/activities/interests as they relate to the <u>current manuscript only</u>.

The author's relationships/activities/interests should be <u>defined broadly</u>. For example, if your manuscript pertains to the epidemiology of hypertension, you should declare all relationships with manufacturers of antihypertensive medication, even if that medication is not mentioned in the manuscript.

In item #1 below, report all support for the work reported in this manuscript without time limit. For all other items, the time frame for disclosure is the past 36 months.

		**Name all entities with whom you have this relationship or indicate none (add rows as needed)**	**Specifications/Comments (e.g., if payments were made to you or to your institution)**
		**Time frame: Since the initial planning of the work**	
1	All support for the present manuscript (e.g., funding, provision of study materials, medical writing, article processing charges, etc.)<br>**No time limit for this item.**	Novo Nordisk, A/S	Novo Nordisk funding of medical writing
		**Time frame: past 36 months**	
2	Grants or contracts from any entity (if not indicated in item #1 above).	__X _None	
3	Royalties or licenses	__X _None	
4	Consulting fees	__X _None	

5	Payment or honoraria for lectures, presentations, speakers bureaus, manuscript writing or educational events	__X _None	
6	Payment for expert testimony	__X _None	
7	Support for attending meetings and/or travel	__X _None	
8	Patents planned, issued or pending	__X _None	
9	Participation on a Data Safety Monitoring Board or Advisory Board	__X _None	
10	Leadership or fiduciary role in other board, society, committee or advocacy group, paid or unpaid	__X _None	
11	Stock or stock options	__X _None	
12	Receipt of equipment, materials, drugs, medical writing, gifts or other services	__X _None	
13	Other financial or non-financial interests	Pentara Corporation	Distinguished Biostatistician

Please place an "X" next to the following statement to indicate your agreement:

X I certify that I have answered every question and have not altered the wording of any of the questions on this form.

ICMJE DISCLOSURE FORM

Date: 01 June, 2026
Your Name: Rikke Mortensen Abschneider
Manuscript Title: Estimating treatment effects in clinical studies of Alzheimer's disease: what are estimands and why do we need them?
Manuscript number (if known): ADJ-D-26-00566

In the interest of transparency, we ask you to disclose all relationships/activities/interests listed below that are related to the content of your manuscript. "Related" means any relation with for-profit or not-for-profit third parties whose interests may be affected by the content of the manuscript. Disclosure represents a commitment to transparency and does not necessarily indicate a bias. If you are in doubt about whether to list a relationship/activity/interest, it is preferable that you do so.

The following questions apply to the author's relationships/activities/interests as they relate to the <u>current manuscript only</u>.

The author's relationships/activities/interests should be <u>defined broadly</u>. For example, if your manuscript pertains to the epidemiology of hypertension, you should declare all relationships with manufacturers of antihypertensive medication, even if that medication is not mentioned in the manuscript.

In item #1 below, report all support for the work reported in this manuscript without time limit. For all other items, the time frame for disclosure is the past 36 months.

		**Name all entities with whom you have this relationship or indicate none (add rows as needed)**	**Specifications/Comments (e.g., if payments were made to you or to your institution)**
		**Time frame: Since the initial planning of the work**	
1	All support for the present manuscript (e.g., funding, provision of study materials, medical writing, article processing charges, etc.) **No time limit for this item.**	Novo Nordisk, A/S	Novo Nordisk funding of medical writing
		**Time frame: past 36 months**	
2	Grants or contracts from any entity (if not indicated in item #1 above).	__X _None	
3	Royalties or licenses	__X _None	
4	Consulting fees	__X _None	
5	Payment or honoraria for lectures, presentations,	__X _None	

	speakers bureaus, manuscript writing or educational events		
6	Payment for expert testimony	__X _None	
7	Support for attending meetings and/or travel	__X _None	
8	Patents planned, issued or pending	__X _None	
9	Participation on a Data Safety Monitoring Board or Advisory Board	__X _None	
10	Leadership or fiduciary role in other board, society, committee or advocacy group, paid or unpaid	__X _None	
11	Stock or stock options	Novo Nordisk, A/S	Minor share holder in Novo Nordisk
12	Receipt of equipment, materials, drugs, medical writing, gifts or other services	__X _None	
13	Other financial or non-financial interests	Novo Nordisk, A/S	Full time employee of Novo Nordisk

Please place an "X" next to the following statement to indicate your agreement:

X I certify that I have answered every question and have not altered the wording of any of the questions on this form.

ICMJE DISCLOSURE FORM

Date: **01 June 2026**
Your Name: **Alireza Atri, MD, PhD**
Manuscript Title: **Estimating treatment effects in clinical studies of Alzheimer's disease: what are estimands and why do we need them?**
Manuscript number (if known): ADJ-D-26-00566

In the interest of transparency, we ask you to disclose all relationships/activities/interests listed below that are related to the content of your manuscript. "Related" means any relation with for-profit or not-for-profit third parties whose interests may be affected by the content of the manuscript. Disclosure represents a commitment to transparency and does not necessarily indicate a bias. If you are in doubt about whether to list a relationship/activity/interest, it is preferable that you do so.

The following questions apply to the author's relationships/activities/interests as they relate to the current manuscript only.

The author's relationships/activities/interests should be defined broadly. For example, if your manuscript pertains to the epidemiology of hypertension, you should declare all relationships with manufacturers of antihypertensive medication, even if that medication is not mentioned in the manuscript.

In item #1 below, report all support for the work reported in this manuscript without time limit. For all other items, the time frame for disclosure is the past 36 months.

		**Name all entities with whom you have this relationship or indicate none (add rows as needed)**	**Specifications/Comments (e.g., if payments were made to you or to your institution)**
		**Time frame: Since the initial planning of the work**	
1	All support for the present manuscript (e.g., funding, provision of study materials, medical writing, article processing charges, etc.) **No time limit for this item.**	Novo Nordisk, A/S	No payment or remuneration to me or my institution for writing or contributing to this manuscript. However, Novo Nordisk provided support for medical writing by employing a medical writing company to provide organizational and medical writing support.
		**Time frame: past 36 months**	
2	Grants or contracts from any entity (if not indicated in item #1 above).	USC, Indiana Univ, Johns Hopkins	Site PI for collaborative clinical trials sponsored or co-sponsored by Universities
		Washington University St. Louis	Project Arm Leader for international clinical trial
		Gates Ventures	Grant from Foundation

		Michael J Fox Foundation (MJFF)	
		AZ DHS, NIA/NIH	Grants from state or federal agency
		Foundation for NIH (FNIH)	PI for Single Site Biomarker (SV2A-PET) study funded by FNIH
		Alzheon, Athira, Biogen	Site PI for biopharma-sponsored clinical trials at institution
		Biohaven (with ADCS),<br>Eisai (with ATRI/ACTC),<br>Lilly (with ATRI/ACTC),<br>Vivoryon (with ADCS)<br>Cognition Therapeutics (with ATRI/ACTC)	Site PI for biopharma-AD academic consortium (ADCS, ACTC, ATRI) collaborative sponsored clinical trials at institution
		ACTC, ADCS,<br>AZ Alzheimer's Research Consortium and AZ DHS,<br>ATRI, GAP	Site PI for clinical trials sponsored or co-sponsored or grants from Academic Research Consortia or Institutes
3	Royalties or licenses	Oxford University Press	Book on dementia
4	Consulting fees	Lundbeck	Past/completed
		Novo Nordisk	Current
		Eisai	Current
		Prothena	Past/completed
		Vaxxinity	Past/completed
		Axsome	Past/completed
		Biogen	Past/completed
		Lantheus	Past/completed
		Life Molecular Imaging	Current
		Qynapse<br>Merck<br>ONO<br>AriBio<br>Johnson & Johnson	Past/completed<br>Past/completed<br>Past/completed<br>Past/completed<br>Past/completed
5	Payment or honoraria for lectures, presentations, speakers bureaus, manuscript writing or educational events	AbbVie<br>Acadia,<br>Allergan,<br>Axovant,<br>AZTherapies,<br>Biogen,<br>Eisai,<br>Lundbeck,<br>Novo Nordisk	Past/completed for all in this category;<br>No speakers bureaus;<br>No payments for manuscript writing
6	Payment for expert testimony	__X _None	
7	Support for attending meetings and/or travel	Alzheimer's Association (US),	Only for workgroup, symposium, summit or consulting mtgs, and scientific/medical presentations or educational programs at conferences or meetings

		Alzheimer's Disease International (ADI), American Academy of Neurology (AAN), American Heart Association (AHA), Michael J Fox Foundation (MJJF), National Academy of Neuropsychology (NAN), International Stroke Conference	
8	Patents planned, issued or pending	__X _None	
9	Participation on a Data Safety Monitoring Board or Advisory Board	Roche/Genentech	Past/completed for Independent Data Safety Monitoring Committee (iDMC)
10	Leadership or fiduciary role in other board, society, committee or advocacy group, paid or unpaid	__X _None	
11	Stock or stock options	__X _None	
12	Receipt of equipment, materials, drugs, medical writing, gifts or other services	__X _None	
13	Other financial or non-financial interests	__X _None	

Please place an "X" next to the following statement to indicate your agreement:

_X__ I certify that I have answered every question and have not altered the wording of any of the questions on this form.

ICMJE DISCLOSURE FORM

Date: 01 June 2026
Your Name: Mette Dahl Bendtsen
Manuscript Title: Estimating treatment effects in clinical studies of Alzheimer's disease: what are estimands and why do we need them?
Manuscript number (if known): ADJ-D-26-00566

In the interest of transparency, we ask you to disclose all relationships/activities/interests listed below that are related to the content of your manuscript. "Related" means any relation with for-profit or not-for-profit third parties whose interests may be affected by the content of the manuscript. Disclosure represents a commitment to transparency and does not necessarily indicate a bias. If you are in doubt about whether to list a relationship/activity/interest, it is preferable that you do so.

The following questions apply to the author's relationships/activities/interests as they relate to the current manuscript only.

The author's relationships/activities/interests should be defined broadly. For example, if your manuscript pertains to the epidemiology of hypertension, you should declare all relationships with manufacturers of antihypertensive medication, even if that medication is not mentioned in the manuscript.

In item #1 below, report all support for the work reported in this manuscript without time limit. For all other items, the time frame for disclosure is the past 36 months.

		**Name all entities with whom you have this relationship or indicate none (add rows as needed)**	**Specifications/Comments (e.g., if payments were made to you or to your institution)**
**Time frame: Since the initial planning of the work**			
1	All support for the present manuscript (e.g., funding, provision of study materials, medical writing, article processing charges, etc.) **No time limit for this item.**	Novo Nordisk, A/S	Novo Nordisk funding of medical writing
**Time frame: past 36 months**			
2	Grants or contracts from any entity (if not indicated in item #1 above).	X None	
3	Royalties or licenses	X None	
4	Consulting fees	X None	

5	Payment or honoraria for lectures, presentations, speakers bureaus, manuscript writing or educational events	X None	
6	Payment for expert testimony	X None	
7	Support for attending meetings and/or travel	X None	
8	Patents planned, issued or pending	X None	
9	Participation on a Data Safety Monitoring Board or Advisory Board	X None	
10	Leadership or fiduciary role in other board, society, committee or advocacy group, paid or unpaid	X None	
11	Stock or stock options	Novo Nordisk	Minor share holder
12	Receipt of equipment, materials, drugs, medical writing, gifts or other services	X None	
13	Other financial or non-financial interests	Novo Nordisk	Employee of Novo Nordisk

Please place an “X” next to the following statement to indicate your agreement:

X I certify that I have answered every question and have not altered the wording of any of the questions on this form.

ICMJE DISCLOSURE FORM

Date: 01 June 2026
Your Name: Teresa Leon Colombo
Manuscript Title: Estimating treatment effects in clinical studies of Alzheimer's disease: what are estimands and why do we need them?
Manuscript number (if known): ADJ-D-26-00566

In the interest of transparency, we ask you to disclose all relationships/activities/interests listed below that are related to the content of your manuscript. "Related" means any relation with for-profit or not-for-profit third parties whose interests may be affected by the content of the manuscript. Disclosure represents a commitment to transparency and does not necessarily indicate a bias. If you are in doubt about whether to list a relationship/activity/interest, it is preferable that you do so.

The following questions apply to the author's relationships/activities/interests as they relate to the current manuscript only.

The author's relationships/activities/interests should be defined broadly. For example, if your manuscript pertains to the epidemiology of hypertension, you should declare all relationships with manufacturers of antihypertensive medication, even if that medication is not mentioned in the manuscript.

In item #1 below, report all support for the work reported in this manuscript without time limit. For all other items, the time frame for disclosure is the past 36 months.

		**Name all entities with whom you have this relationship or indicate none (add rows as needed)**	**Specifications/Comments (e.g., if payments were made to you or to your institution)**
		**Time frame: Since the initial planning of the work**	
1	All support for the present manuscript (e.g., funding, provision of study materials, medical writing, article processing charges, etc.) **No time limit for this item.**	Novo Nordisk, A/S	Novo Nordisk funding of medical writing.
		**Time frame: past 36 months**	
2	Grants or contracts from any entity (if not indicated in item #1 above).	__X _None	
3	Royalties or licenses	__X _None	
4	Consulting fees	__X _None	
5	Payment or honoraria for lectures, presentations, speakers bureaus,	__X _None	

	manuscript writing or educational events		
6	Payment for expert testimony	__X _None	
7	Support for attending meetings and/or travel	__X _None	
8	Patents planned, issued or pending	__X _None	
9	Participation on a Data Safety Monitoring Board or Advisory Board	Novo Nordisk, A/S	Organized advisory boards
10	Leadership or fiduciary role in other board, society, committee or advocacy group, paid or unpaid	__X _None	
11	Stock or stock options	Novo Nordisk, A/S	Minor share holder
		PharmaMar	Minor share holder
12	Receipt of equipment, materials, drugs, medical writing, gifts or other services	__X _None	
13	Other financial or non-financial interests	Novo Nordisk, A/S	Employee of Novo Nordisk

Please place an "X" next to the following statement to indicate your agreement:

X I certify that I have answered every question and have not altered the wording of any of the questions on this form.

ICMJE DISCLOSURE FORM

Date: 01 June 2026
Your Name: Howard H. Feldman
Manuscript Title: Estimating treatment effects in clinical studies of Alzheimer's disease: what are estimands and why do we need them?
Manuscript number (if known): ADJ-D-26-00566

In the interest of transparency, we ask you to disclose all relationships/activities/interests listed below that are related to the content of your manuscript. "Related" means any relation with for-profit or not-for-profit third parties whose interests may be affected by the content of the manuscript. Disclosure represents a commitment to transparency and does not necessarily indicate a bias. If you are in doubt about whether to list a relationship/activity/interest, it is preferable that you do so.

The following questions apply to the author's relationships/activities/interests as they relate to the current manuscript only.

The author's relationships/activities/interests should be defined broadly. For example, if your manuscript pertains to the epidemiology of hypertension, you should declare all relationships with manufacturers of antihypertensive medication, even if that medication is not mentioned in the manuscript.

In item #1 below, report all support for the work reported in this manuscript without time limit. For all other items, the time frame for disclosure is the past 36 months.

		Name all entities with whom you have this relationship or indicate none (add rows as needed)	Specifications/Comments (e.g., if payments were made to you or to your institution)
		**Time frame: Since the initial planning of the work**	
1	All support for the present manuscript (e.g., funding, provision of study materials, medical writing, article processing charges, etc.) **No time limit for this item.**	Novo Nordisk, A/S	Novo Nordisk funding to OPEN Health Communications for medical writing support in accordance with Good Publication guidelines. No funding to HF or his institution for this publication.
		**Time frame: past 36 months**	
2	Grants or contracts from any entity (if not indicated in item #1 above).	Allyx Therapeutics	Grant funding to UC San Diego
		Vivoryon Therapeutics (Probiodrug)	Grant funding to UC San Diego
3	Royalties or licenses	Feldman HH (filed November 26, 2008). Detecting and Treating Dementia Serial Number 12/3-2691 U.S. Patent No. PCT/US2007/07008. Washington, DC: U.S. Patent and Trademark Office	
4	Consulting fees	LuMind Foundation	Service agreement with UC San Diego for consulting activities
		Axon Neuroscience	Service agreement with UC San Diego for consulting activities

		Arrowhead Pharmaceuticals	Service agreement with UC San Diego for consulting activities
		Biosplice Therapeutics, Inc.	Service agreement with UC San Diego for consulting activities
		Novo Nordisk, Inc	Service agreement with UC San Diego for consulting activitiies
5	Payment or honoraria for lectures, presentations, speakers bureaus, manuscript writing or educational events	X None	
6	Payment for expert testimony	X None	
7	Support for attending meetings and/or travel	Royal Society of Canada	Payment to UC San Diego for travel related expenses for conference presentation, November 2023
		Translating Research in Elder Care (TREC)	Payment to UC San Diego for travel related expenses for conference presentation, June 2024
		Association for Frontotemporal Dementia (AFTD)	Payment to UC San Diego for travel related expenses at research roundtable, September 2023, August 2024, September 2025
		Rainwater Charitable Foundation	Payment to UC San Diego for travel related expenses to Tau Consortium SAB and Rainwater Prize meetings
		Banner Health	Payment to UC San Diego for travel related expenses to participate in conference on the prevention of AD, Feb 2025
		Invictus	Payment to UC San Diego for travel related expenses to present at the 15th Turkish Alzheimer Congress in Antalya, Oct 2026
		Summeet	Payment to UC San Diego for travel related expenses to present at the Change in Cardiology Congress in Turin, Italy, April 2026
		Novo Nordisk, Inc.	Payment for travel related expenses to present evoke+ results at the 20th international conference on AD/PD in Copenhagen, March 2026.
8	Patents planned, issued or pending	Feldman HH (filed November 26, 2008). Detecting and Treating Dementia Serial Number 12/3-2691 U.S. Patent No. PCT/US2007/07008. Washington, DC: U.S. Patent and Trademark Office	
9	Participation on a Data Safety Monitoring Board or Advisory Board	Roche/Genentech Pharmaceuticals	Service agreement with UC San Diego for DMC and DSMB
		Tau Consortium Rainwater Charitable Foundation	Service agreement with UC San Diego for Scientific Advisory Board Chair
		Janssen Research & Development LLC	Service agreement with UC San Diego for DSMB
10	Leadership or fiduciary role in other board, society, committee or advocacy group, paid or unpaid	X None	
11	Stock or stock options	X None	
12	Receipt of equipment, materials, drugs, medical	X None	

	writing, gifts or other services		
13	Other financial or non-financial interests	Epstein Family Alzheimer's Research Collaboration	Philanthropic support for Alzheimer therapeutic research

Please place an "X" next to the following statement to indicate your agreement:

X I certify that I have answered every question and have not altered the wording of any of the questions on this form.

ICMJE DISCLOSURE FORM

Date: 01 June 2026
Your Name: Peter Johannsen
Manuscript Title: Estimating treatment effects in clinical studies of Alzheimer's disease: what are estimands and why do we need them?
Manuscript number (if known): ADJ-D-26-00566

In the interest of transparency, we ask you to disclose all relationships/activities/interests listed below that are related to the content of your manuscript. "Related" means any relation with for-profit or not-for-profit third parties whose interests may be affected by the content of the manuscript. Disclosure represents a commitment to transparency and does not necessarily indicate a bias. If you are in doubt about whether to list a relationship/activity/interest, it is preferable that you do so.

The following questions apply to the author's relationships/activities/interests as they relate to the current manuscript only.

The author's relationships/activities/interests should be defined broadly. For example, if your manuscript pertains to the epidemiology of hypertension, you should declare all relationships with manufacturers of antihypertensive medication, even if that medication is not mentioned in the manuscript.

In item #1 below, report all support for the work reported in this manuscript without time limit. For all other items, the time frame for disclosure is the past 36 months.

		**Name all entities with whom you have this relationship or indicate none (add rows as needed)**	**Specifications/Comments (e.g., if payments were made to you or to your institution)**
		**Time frame: Since the initial planning of the work**	
1	All support for the present manuscript (e.g., funding, provision of study materials, medical writing, article processing charges, etc.) **No time limit for this item.**	Novo Nordisk, A/S	Novo Nordisk funding of medical writing
		**Time frame: past 36 months**	
2	Grants or contracts from any entity (if not indicated in item #1 above).	_x___None	
3	Royalties or licenses	_x___None	
4	Consulting fees	_x___None	
5	Payment or honoraria for lectures, presentations, speakers bureaus,	__x__None	

	manuscript writing or educational events		
6	Payment for expert testimony	__x__None	
7	Support for attending meetings and/or travel	__x__None	
8	Patents planned, issued or pending	__x__None	
9	Participation on a Data Safety Monitoring Board or Advisory Board	__x__None	
10	Leadership or fiduciary role in other board, society, committee or advocacy group, paid or unpaid	__x__None	
11	Stock or stock options	Novo Nordisk, A/S	Minor shareholder of Novo Nordisk
12	Receipt of equipment, materials, drugs, medical writing, gifts or other services	_x___None	
13	Other financial or non-financial interests	Novo Nordisk, A/S	Employee of Novo Nordisk

Please place an "X" next to the following statement to indicate your agreement:

_X__ I certify that I have answered every question and have not altered the wording of any of the questions on this form.

ICMJE DISCLOSURE FORM

Date: 01 June 2026
Your Name: Jeffrey Cummings
Manuscript Title: Estimating treatment effects in clinical studies of Alzheimer's disease: what are estimands and why do we need them?
Manuscript number (if known): ADJ-D-26-00566

In the interest of transparency, we ask you to disclose all relationships/activities/interests listed below that are related to the content of your manuscript. "Related" means any relation with for-profit or not-for-profit third parties whose interests may be affected by the content of the manuscript. Disclosure represents a commitment to transparency and does not necessarily indicate a bias. If you are in doubt about whether to list a relationship/activity/interest, it is preferable that you do so.

The following questions apply to the author's relationships/activities/interests as they relate to the current manuscript only.

The author's relationships/activities/interests should be defined broadly. For example, if your manuscript pertains to the epidemiology of hypertension, you should declare all relationships with manufacturers of antihypertensive medication, even if that medication is not mentioned in the manuscript.

In item #1 below, report all support for the work reported in this manuscript without time limit. For all other items, the time frame for disclosure is the past 36 months.

		**Name all entities with whom you have this relationship or indicate none (add rows as needed)**	**Specifications/Comments (e.g., if payments were made to you or to your institution)**
**Time frame: Since the initial planning of the work**			
1	All support for the present manuscript (e.g., funding, provision of study materials, medical writing, article processing charges, etc.) **No time limit for this item.**	Novo Nordisk, A/S	Novo Nordisk funding of medical writing
**Time frame: past 36 months**			
2	Grants or contracts from any entity (if not indicated in item #1 above).	NIGMS grant P20GM109025; NIA R35AG71476; NIA R25AG083721; NINDS RO1NS139383; Alzheimer's Disease Drug Discovery Foundation (ADDF); Ted and Maria Quirk Endowment; Joy Chambers-Grundy Endowment.	Payments made to my institution

3	Royalties or licenses	Neuropsychiatric Inventory	Copyright fees paid to me
4	Consulting fees	Acadia, Alkermes, ALZpath, AnnovisBio, Artery, Axsome, Biogen, Bristol-Myers Squib, Eisai, Fosun, GAP Foundation, IGC, Janssen, Julius Clinical, Kinoxis, Lilly, Lundbeck, LSP/eqt, Merck (Merck, Sharp, and Dohme), MoCA Cognition, Novo Nordisk, NSC Therapeutics, Otsuka, ReMYND, Roche, Scottish Brain Sciences, Signant Health, Simcere, and sinaptica therapeutics	Paid to me as consultant
5	Payment or honoraria for lectures, presentations, speakers bureaus, manuscript writing or educational events	__X _None	
6	Payment for expert testimony	__X _None	
7	Support for attending meetings and/or travel	__X _None	
8	Patents planned, issued or pending	__X _None	
9	Participation on a Data Safety Monitoring Board or Advisory Board	__X _None	
10	Leadership or fiduciary role in other board, society, committee or advocacy group, paid or unpaid	Global Alzheimer Platform	Uncompensated board member
11	Stock or stock options	ALZpath; Beren	Unrealized stock options
12	Receipt of equipment, materials, drugs, medical writing, gifts or other services	__X _None	
13	Other financial or non-financial interests	__X _None	

Please place an "X" next to the following statement to indicate your agreement:

_X__ I certify that I have answered every question and have not altered the wording of any of the questions on this form.
